# Next-generation of targeted AAVP vectors for systemic transgene delivery against cancer

**DOI:** 10.1073/pnas.1906653116

**Published:** 2019-08-02

**Authors:** Keittisak Suwan, Teerapong Yata, Sajee Waramit, Justyna M. Przystal, Charlotte A. Stoneham, Kaoutar Bentayebi, Paladd Asavarut, Aitthiphon Chongchai, Peraphan Pothachareon, Koon-Yang Lee, Supachai Topanurak, Tracey L. Smith, Juri G. Gelovani, Richard L. Sidman, Renata Pasqualini, Wadih Arap, Amin Hajitou

**Affiliations:** ^a^Phage Therapy Group, Department of Brain Sciences, Imperial College London, W12 0NN London, United Kingdom;; ^b^Thailand Excellence Center for Tissue Engineering and Stem Cells, Department of Biochemistry, Faculty of Medicine, Chiang Mai University, 50200 Chiang Mai, Thailand;; ^c^Department of Aeronautics, Imperial College London, SW7 2AZ London, United Kingdom;; ^d^Department of Molecular Tropical Medicine and Genetics, Faculty of Tropical Medicine, Mahidol University, 10400 Bangkok, Thailand;; ^e^Rutgers Cancer Institute of New Jersey, Newark, NJ 07103;; ^f^Division of Cancer Biology, Department of Radiation Oncology, Rutgers New Jersey Medical School, Newark, NJ 07103;; ^g^Karmanos Cancer Institute, School of Medicine, Wayne State University, Detroit, MI 48201;; ^h^Department of Biomedical Engineering, College of Engineering, Wayne State University, Detroit, MI 48201;; ^i^Department of Neurology, Harvard Medical School, Boston, MA 02115;; ^j^Division of Hematology/Oncology, Department of Medicine, Rutgers New Jersey Medical School, Newark, NJ 07103

**Keywords:** AAVP, cancer, gene delivery, phage display, preclinical studies

## Abstract

Bacteriophage (phage) have attractive advantages as delivery systems compared with mammalian viruses, but have been considered poor vectors because they lack evolved strategies to confront and overcome mammalian cell barriers to infective agents. We reasoned that improved efficacy of delivery might be achieved through structural modification of the viral capsid to avoid pre- and postinternalization barriers to mammalian cell transduction. We generated multifunctional hybrid adeno-associated virus/phage (AAVP) particles to enable simultaneous display of targeting ligands on the phage’s minor pIII proteins and also degradation-resistance motifs on the very numerous pVIII coat proteins. This genetic strategy of directed evolution bestows a next-generation of AAVP particles that feature resistance to fibrinogen adsorption or neutralizing antibodies and ability to escape endolysosomal degradation. This results in superior gene transfer efficacy in vitro and also in preclinical mouse models of rodent and human solid tumors. Thus, the unique functions of our next-generation AAVP particles enable improved targeted gene delivery to tumor cells.

## Significance

Over the past decade, we have used adeno-associated virus phage (AAVP) vectors for discovery, preclinical imaging, and translation therapy. However, hurdles remained with AAVP-mediated transgene delivery: both preinternalization (nonspecific protein adsorption, resulting in antibody-based neutralization) and postinternalization (endolysosomal degradation). As a proof-of-concept, we designed, developed, and validated a next-generation of targeted AAVP vectors that mitigate these obstacles to efficient gene delivery through the *cis*-genetic incorporation of specific peptide motifs to the phage capsid. On direct testing, the next-generation of targeted AAVP vectors proved superior to the original prototype in vitro and in mouse models of solid tumors, by avoiding degradation in pre- and postinternalization settings: An important methodological advance. We propose this platform should be considered for clinical applications.

Bacteriophage (phage) are viruses that infect and replicate only within 1 or another bacterial hosts. A major advantage of ligand peptide-targeted phage-based vectors—as opposed to mammalian viruses—for gene delivery to human diseases is that phages internalize into nonbacterial cells only if they are genetically modified to do so (1, 2). However, phage vectors are often deemed poor for gene transfer into mammalian cells, the gene delivery depending on a vector’s ability to resist a harsh vascular microenvironment containing plasma proteins causing nonspecific adsorption and antibody neutralization. The phage must bind to a corresponding receptor on the membrane surface of target cells and then internalize there (3). Once internalized, the vector has to escape lysosomal degradation and be released into the cytoplasm (4). Finally, the vector must “uncoat” and pass from the cytoplasm into the nucleus, where the formerly exogenous gene (now called a “transgene”) is to be expressed in its new target cell (5).

In extensive previous work (6–18), we showed that the efficiency of transgene delivery and expression is markedly improved if the mammalian genes are incorporated in *cis* form, such as inverted terminal repeats (ITR) from the human adeno-associated virus (AAV), into the phage genome background (9, 10). However, our first generation of targeted AAV/phage (AAVP) hybrid vectors often failed to overcome inherent obstacles to efficient transgene expression in targeted mammalian cells, such as by avoiding plasma protein nonspecific adsorption or by enabling endolysosomal escape from degradation (19), while maintaining their other desirable functional attributes. Therefore, we sought opportunities to address such unmet needs by incorporating capsid display elements that could overcome barriers to viral particle preinternalization, homing, and postinternalization trafficking, and thereby improve targeted transgene expression in mammalian—especially human—cells of interest.

Here, we introduce next-generation targeted AAVP constructs that either mitigate or eliminate 2 of the major mammalian cell barriers to transgene delivery and expression, subsequently resulting in improved transgene delivery under in vitro and in vivo conditions in preclinical cancer models. Given their superior profile relative to our earlier AAVP vectors, these improved targeted constructs are likely to become standard vectors of choice for translational development.

## Results

### Conception, Generation, and Characterization of a Next-Generation Multifunctional Display System.

**Fig. 1.**
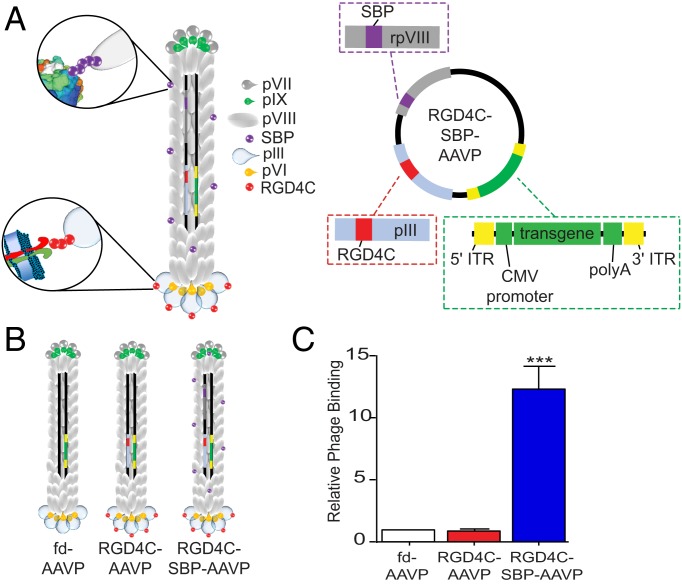
Schematic representation of a multifunctional AAVP targeting system. (*A*) The multifunctional AAVP displays RGD4C peptide ligands on the pIII minor coat protein at 1 end of the phage, and multiple copies of SBP on the rpVIII major protein coating the surface of the phage. Moreover, a mammalian transgene cassette, driven by the cytomegalovirus (CMV) promoter and flanked by AAV ITRs, was inserted into the single-stranded genome located internally in the phage, serving for transgene expression. (*B*) Schematic representation of control and multifunctional AAVP constructs. (*C*) Streptavidin-binding capacity. The different AAVP constructs were incubated with immobilized streptavidin and washed before infection of K91Kan *E. coli*. Data represent the mean ± SEM of triplicate samples from 1 representative experiment of 3. ***P < 0.001.

To conceptualize and generate a multifunctional AAVP capable of displaying additional ligands on the major pVIII protein, we engineered a phage genome that bears 2 versions of the M13 single-stranded gene VIII, encoding 2 different types of pVIII molecules: Wild type (WT) and recombinant. Other phage-based constructs have been designed to exploit the functional display of ligand peptides on the pVIII major coat proteins for various purposes, including crossing cellular membranes (20–23). The new generation of AAVP are composed of both WT and recombinant pVIII (rpVIII) subunits, and the resulting multifunctional hybrid constructs are able to: (1) display the targeting ligand on the phage pIII minor coat protein for binding to a mammalian receptor, (2) display foreign functional peptides on the WT pVIII or rpVIII major coat proteins, and (3) bear a mammalian transgene cassette inserted in an intergenomic region of the phage genome for gene expression in mammalian cells. To construct a multifunctional hybrid AAVP, the genomes of 2 existing M13 filamentous phage that share similar genetic backbones were combined. The fUSE5 genome bears a single gene III to display the targeting peptide, and the f88.4 genome contains 2 gene VIIIs encoding both WT pVIII and rpVIII. The tumor-targeting double-cyclic ligand RGD4C (CDCRGDCFC) peptide (24–26) was incorporated into the minor coat protein of fUSE5 and a chimera with f88.4 was constructed before introducing additional genetic sequences encoding the desired peptides in different coat protein genes by use of a subcloning strategy and site-directed mutagenesis. We have also introduced a mammalian transgene cassette flanked by AAV2 ITRs in a phage intergenomic region to generate an AAVP for targeted gene delivery. To evaluate the principle initially, we first designed an AAVP displaying a well-characterized streptavidin-binding peptide (SBP). The genetic sequence encoding SBP (peptide ANRLCHPQFPCTSHE) was fused in-frame with the rpVIII gene (20). Thus, in addition to the mammalian transgene cassette, the resulting particle simultaneously displayed an RGD4C ligand at the phage terminus and multiple copies of SBP on the surface, as schematically shown in Fig. 1*A*. All constructs were analyzed by restriction enzyme digest to map enzyme and DNA sequencing, confirming the correct orientation of the inserts and ruling out any mutations that might have occurred during the cloning steps. The resulting RGD4C-SBP-AAVP and controls are schematically shown in Fig. 1*B*.

**Fig. 2.**
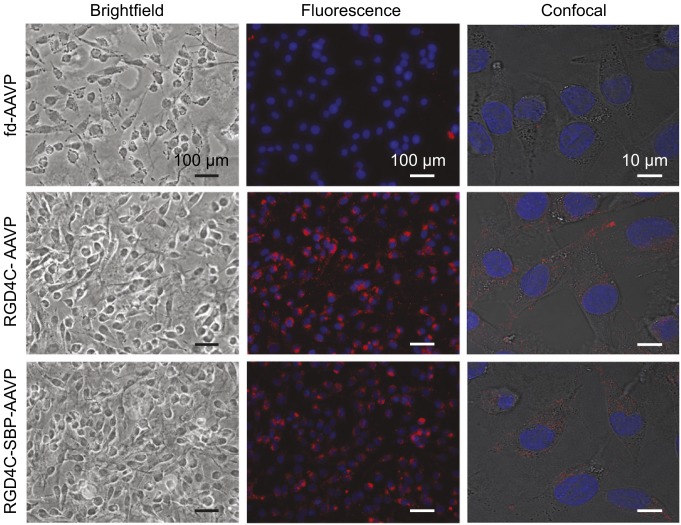
Binding and internalization of AAVP vectors. Immunofluorescence imaging was performed on cultured human M21 melanoma cells following incubation with different AAVP constructs (10^6^ TU per cell). Internalized AAVP particles were labeled with antiphage antibody (red) and nuclei were stained with DAPI (blue). Confocal imaging of immunofluorescence-based antiphage staining of M21 cells was also done (Scale bars, 10 or 100 μm.)

To demonstrate that the SBP moieties displayed on the rpVIII coat proteins were functional, we performed an in vitro binding assay on a streptavidin-coated plate. Unbound particles were removed from the plate through a series of washes, and bound AAVP particles were recovered by infection of *Escherichia coli* K91 host bacteria. We found that the multifunctional AAVP displaying SBP (RGD4C-SBP-AAVP) bound to immobilized streptavidin as determined by the greater number of bacterial transducing units (TU). In contrast, corresponding negative control constructs (namely, RGD4C-AAVP and nontargeted control fd-AAVP, which lacks a targeting ligand on the phage pIII) did not show any detectable binding above background (Fig. 1*C*). We also validated the function of the RGD4C targeting ligand displayed on the pIII minor coat protein by assessing the binding and internalization to cells expressing the αvβ3 integrins. Immunofluorescence and confocal microscopy with antibodies against the phage capsid were performed on M21 cells, which are known to express high levels of αvβ3 integrins (27). As shown in Fig. 2, the targeting and internalization capabilities of the RGD4C peptide remained functionally intact in RGD4C-AAVP and RGD4C-SBP-AAVP; the nontargeted control fd-AAVP showed background signal only. Taken together, these results establish the conceptual feasibility of the multifunctional AAVP particles.

### A Multifunctional AAVP Containing a WT Major Coat pVIII Protein with Altered Charge.

**Fig. 3.**
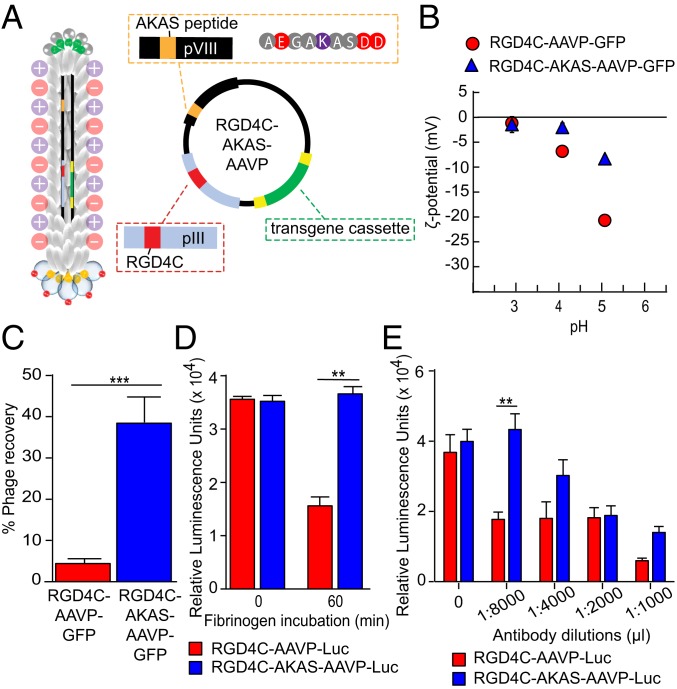
The AKAS motif displayed on the WT pVIII bestows unique physical features of the multifunctional AAVP. (*A*) Design of the RGD4C-AKAS-AAVP. (*B*) ζ-Potential was measured by Zetasizer as a function of pH. Data represent the mean ± SEM of triplicate samples from 1 representative experiment of 3. (*C*) The cationic polymer binding capacity of multifunctional RGD4C-AKAS-AAVP-*GFP* was tested. The amount of AAVP recovered from the DEAE.DEX-coated plate is reported as a percentage of input. (*D*) AAVP resistance to fibrinogen was evaluated. 9L cells were transduced with 3 × 10^4^ TU per cell AAVP for 4 h in serum-free media before (time 0) or after 60-min incubation with fibrinogen. (*E*) The 9L cells were incubated with 3 × 10^4^ TU per cell AAVP in media containing antiphage antibody in the indicated dilutions. Two experiments were performed, and the results represent the mean ± SEM relative luminescence units (RLU)/100 μg of protein from triplicate wells, at 3 d posttransduction. **P < 0.01; ***P < 0.001.

After confirming that ligand peptides displayed on the multifunctional AAVP retain their binding attributes, we sought to investigate whether a similar construct could be used to overcome extracellular barriers and to improve gene transfer subsequently in the presence of these obstacles. The negative charge of the M13 phage surface induces high levels of nonspecific extracellular binding to positively charged molecules (∼35% of proteins in the human proteome). Thus, a major extracellular barrier to systemic gene delivery vectors is the formation of a “protein corona” caused by nonspecific plasma protein adsorption to vectors (28). Therefore, we have reasoned that a potential way to avoid this problem would be to introduce zwitterionic properties, known for being resistant to plasma protein adsorption, onto the viral particle surface (29). As such, we have designed a multifunctional AAVP to change the negative N terminus of the major WT pVIII coat protein into a mixture of anionic and cationic terminal groups by introducing a short charged neutralizing peptide with the sequence Ala-Lys-Ala-Ser or AKAS (Fig. 3*A*). This unique targeted multifunctional AAVP vector (termed RGD4C-AKAS-AAVP) should display zwitterionic properties. A general feature of zwitterionic materials is that they possess both positively and negatively charged moieties on the same side chain, thereby either decreasing total negative charge or achieving neutrality (30–32). To investigate whether the AKAS peptide altered the negatively charged AAVP capsid, we measured the ζ-potential by electrophoresis. As predicted, we found that AAVP is negatively charged at acidic pH (Fig. 3*B*). The data also indicated that RGD4C-AAVP possesses an acidic surface, with an isoelectric point of pH = 3. Notably, we found a substantial shift of the ζ-potential of the multifunctional RGD4C-AKAS-AAVP toward lower negative values, moving the ζ-potential from −20.71 mV to −8.32 mV. Such alteration in ζ-potential is demonstrably attributed to the zwitterionic property of the modified AAVP particles displaying the AKAS peptide, consistent with our original hypothesis.

To confirm the zwitterionic attributes of RGD4C-AKAS-AAVP and to demonstrate that the modified multifunctional particle surface has different charge properties compared with the parental AAVP, we measured the cationic polymer binding capacity of phage. We incubated the AAVP with the positively charged DEAE.DEX polymer and recovered unbound particles after overnight incubation. As shown in Fig. 3*C*, a minimal number of RGD4C-AAVP was recovered compared with the RGD4C-AKAS-AAVP, suggesting that the original vector was almost completely sequestered by the DEAE.DEX polymers. Interestingly, ∼40% of the RGD4C-AKAS-AAVP was recovered (Fig. 3*C*), demonstrating that the surface of the newly generated AAVP construct was successfully modified through genetic engineering to reduce the binding capacity to positively charged molecules.

### RGD4C-AKAS-AAVP Features Resistance to Fibrinogen Adsorption and to Antibody Neutralization.

Fibrinogen is extensively used as a model protein to assess the protein adsorption resistance of biomaterials (33). To investigate the effect of a fibrinogen barrier on gene transfer efficacy, we tested the protein resistance property of RGD4C-AKAS-AAVP on gene transfer to cells following AAVP vector incubation with fibrinogen proteins. Rat 9L glioma cells, which express αvβ3 integrins, were treated with the parental RGD4C-AAVP-*Luc* or RGD4C-AKAS-AAVP-*Luc* (each carrying a *luciferase* reporter gene) under normal conditions or following incubation with fibrinogen for 60 min (Fig. 3*D*). No differences in luciferase expression were detected in normal conditions. Interestingly, after incubation with fibrinogen, a 2.2-fold decrease in luciferase expression was observed in tumor cells transduced with RGD4C-AAVP-*Luc* compared with cells treated with RGD4C-AKAS-AAVP-*Luc* (Fig. 3*D*). This result indicates that multifunctional AAVP with AKAS-altered surface is able to minimize or avoid nonspecific binding to fibrinogen.

To uncover further potential advantages acquired by the multifunctional RGD4C-AKAS-AAP-*Luc*, we evaluated the effect of a neutralizing antibody that recognizes the coat proteins of the parental phage capsid on the transduction efficiency of the multifunctional AAVP. Tumor 9L cells were treated with either RGD4C-AAVP-*Luc* or RGD4C-AKAS-AAVP-*Luc* in the presence of antiphage antibody and luciferase activity was determined 3 d posttransduction (Fig. 3*E*). The data showed a clear difference in gene expression in the presence of antiphage antibody. Indeed, we observed 2.5-fold higher transduction efficiency by RGD4C-AKAS-AAVP-*Luc*, in the presence of a particular antibody concentration (1:8,000) compared with cells transduced with RGD4C-AAVP-*Luc* (Fig. 3*E*). These data indicate that the AKAS-modified multifunctional AAVP particles acquired an escape mechanism to avoid antibody-mediated neutralization.

### Design and Validation of a Multifunctional AAVP to Escape Endosomal Sequestration.

**Fig. 4.**
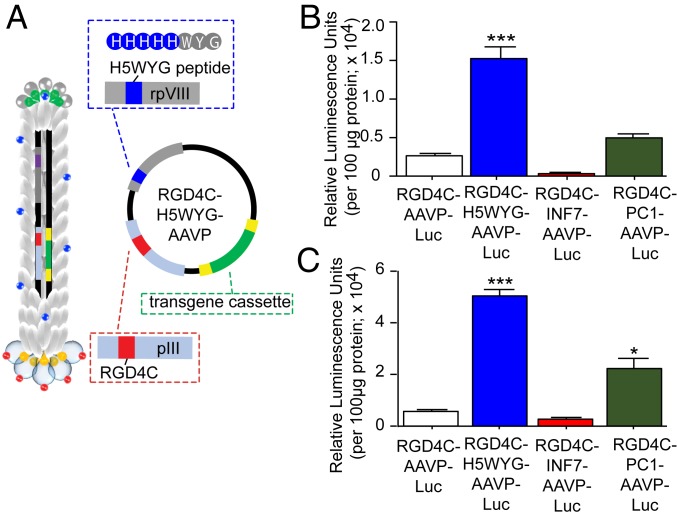
Multifunctional AAVP displaying endosome-escape peptides. (*A*) Schematic representation of RGD4C targeted multifunctional AAVP displaying the H5WYG endosome-escape peptide motif. (*B*) Human M21 melanoma cells or (*C*) rat 9L glioma cells were treated with 3 × 10^4^ TU per cell of RGD4C-targeted multifunctional AAVP displaying the endosome-escape peptides H5WYG, INF7, or PC1. Luciferase expression was measured at 3 d posttransduction. Luciferase activity was expressed as mean RLU normalized to protein amount. *P < 0.05; ***P < 0.001.

We have previously identified the endosomal-lysosomal degradative pathway as a major intracellular limitation to targeted RGD4C-AAVP, as the particles are sequestered and degraded within the lysosomes, greatly decreasing their ability to deliver genes to mammalian cells (19); those studies were performed by pharmacological approaches with chloroquine or bafilomycin A1 as transdisruptive agents. In contrast, we engineered here 3 multifunctional AAVP displaying peptides on the rpVIII coat proteins to potentially promote escape from the endo-lysosomal degradative pathway. First, the H5WYG peptide (sequence GLFHAIAHFIHGGWHGLIHGWYG) is a histidylated fusogenic peptide with endosomal buffering capacity, derived from the N-terminal sequence of the HA2 subunit of the influenza virus hemagglutinin (34) (Fig. 4*A*). INF7 (a 23-mer acidic derivative) is a pH-dependent fusogenic peptide derived from the N terminal of influenza HA2 with high specificity for low pH (5.5) and higher membrane lytic activity (35). Finally, the PC1 peptide has a pH-dependent endosomolytic sequence identified by phage display; due to its tryptophan content, it was proposed that the peptide may cause endosomal membrane lysis by insertion into lipid bilayers (36). To assess gene transduction efficacy, we administered the multifunctional AAVP constructs carrying the *luciferase* reporter gene to M21 human melanoma cells and measured the total luciferase expression from each vector relative to the corresponding controls. The targeted multifunctional AAVP displaying the H5WYG peptide (RGD4C-H5WYG-AAVP-*Luc*) resulted in substantially more gene expression compared with the other constructs and RGD4C-AAVP-*Luc*, which lacks any modification to pVIII. (Fig. 4*B*). To rule out the possibility that the observed effects might have been either cell- or species-specific, we repeated these experiments in rat 9L glioma cells and found a similar pattern of clearly increased gene transduction by the multifunctional RGD4C-H5WYG-AAVP-*Luc* (Fig. 4*C*). For example, luciferase expression levels from the RGD4C-H5WYG-AAVP-*Luc* vector reached ∼6- and ∼9-fold higher than expression from RGD4C-AAVP-*Luc* in M21 and 9L cells, respectively.

We performed additional experiments to evaluate transgene expression following mammalian cell transduction with various genomic copies (GC) of the multifunctional AAVP carrying the *luciferase* reporter gene, compared with the previously described RGD4C-AAVP-*Luc* construct, in both M21 and 9L cells. After PCR analyses to quantify GC, we added serially increasing GC per cell, ranging from 10^3^ to 10^7^ GC per cell, and measured luciferase expression 3 d posttransduction. The data showed that transduction by the multifunctional RGD4C-H5WYG-AAVP-*Luc* or by RGD4C-AAVP-*Luc* increased with increasing vector GC per cell (*SI Appendix*, Fig. S1). Furthermore, in these experiments, the multifunctional RGD4C-H5WYG-AAVP-*Luc* proved superior in targeted gene delivery compared with RGD4C-AAVP-*Luc* for all GC per cell doses, in both M21 and 9L tumor cells (*SI Appendix*, Fig. S1). Of note, no gene expression was detected in M21 and 9L cells given nontargeted control fd-H5WYG-AAVP-*Luc*, confirming that targeted transduction of cancer cells by AAVP is markedly improved with the multifunctional RGD4C-H5WYG-AAVP-*Luc*.

### Endosomal Escape and Buffering Capacity of the Multifunctional RGD4C-H5WYG-AAVP.

**Fig. 5.**
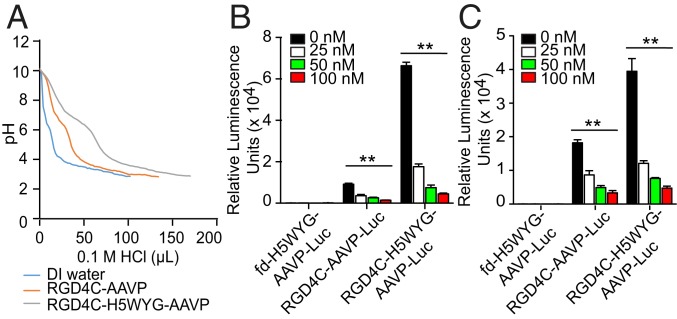
Endosome buffering capacity of AAVP. (*A*) Acid–base titration curve of AAVP. AAVP particles were dissolved in water and adjusted to pH 10. Multifunctional RGD4C-H5WYG-AAVP-*Luc* and RGD4C-AAVP-*Luc*, which has no peptides displayed on rpVIII, were included in this experiment. HCl was used to titrate the solutions to pH = 3 since the typical pH range in the endosome is pH 7.0 to 4.0. A titration curve of water was done as a control. (*B* and *C*) Effect of bafilomycin A1 on transduction efficiency. Either (*B*) M21 or (*C*) 9L tumor cells were treated with increasing concentrations of bafilomycin A1 for 1 h, then incubated with 3 × 10^4^ TU per cell of either RGD4C-H5WYG-AAVP-*Luc*, RGD4C-AAVP-*Luc*, or nontargeted control fd-H5WYG-AAVP-*Luc*. After 3 d, cells were analyzed for luciferase expression. **P < 0.01.

We next sought to confirm that increased gene delivery by RGD4C-H5WYG-AAVP is associated with vector escape from the endosomes. Histidine-rich peptides are thought to promote endosomal escape through protonation of their imidazole groups in the acidic conditions of the endosomes, which may result in osmotic swelling, rupture, and release of the endosomal contents, termed the “proton-sponge effect” (37). The buffering capacity of the H5WYG peptide should allow the RGD4C-H5WYG-AAVP to absorb protons pumped into the endosomes, resulting in an influx of Cl^−^ ions to prevent the build-up of a charge gradient. This influx of both protons and Cl^−^ ions increases the osmolarity of the endosomes that leads to osmotic swelling, subsequent destabilization, and release of their contents into the cytoplasm. To assess the buffering capacity of the RGD4C-H5WYG-AAVP, we performed acid–base titrations of RGD4C-H5WYG-AAVP, RGD4C-AAVP lacking the H5WYG peptide on rpVIII, or aqueous vehicle as controls. We found that the RGD4C-H5WYG-AAVP had a markedly higher buffering capacity compared with RGD4C-AAVP and required additional HCl to lower the pH from 7.0 to 4.0 (Fig. 5*A*). These data provide evidence that the increased buffering capacity of RGD4C-H5WYG-AAVP contributes to its improved gene delivery efficiency and gives credence to the hypothesis that the modifications to rpVIII on the multifunctional AAVP facilitate particle escape from the endo-lysosomal degradative pathway. To confirm these findings, we performed transduction experiments in the presence of bafilomycin A1, a specific inhibitor of the vacuolar ATPase proton pump, that prevents endosomal escape, and tested increasing and nontoxic concentrations of bafilomycin A1, 0 to 100 nM (38). The results showed a substantial decrease in gene delivery efficacy of RGD4C-H5WYG-AAVP in the presence of bafilomycin A1, in a concentration-dependent manner in both tumor cell types, M21 melanoma (Fig. 5*B*) and 9L glioma (Fig. 5*C*).

### RGD4C-H5WYG-AAVP Promotes Targeted Gene Delivery to Solid Tumors in Preclinical Models.

**Fig. 6.**
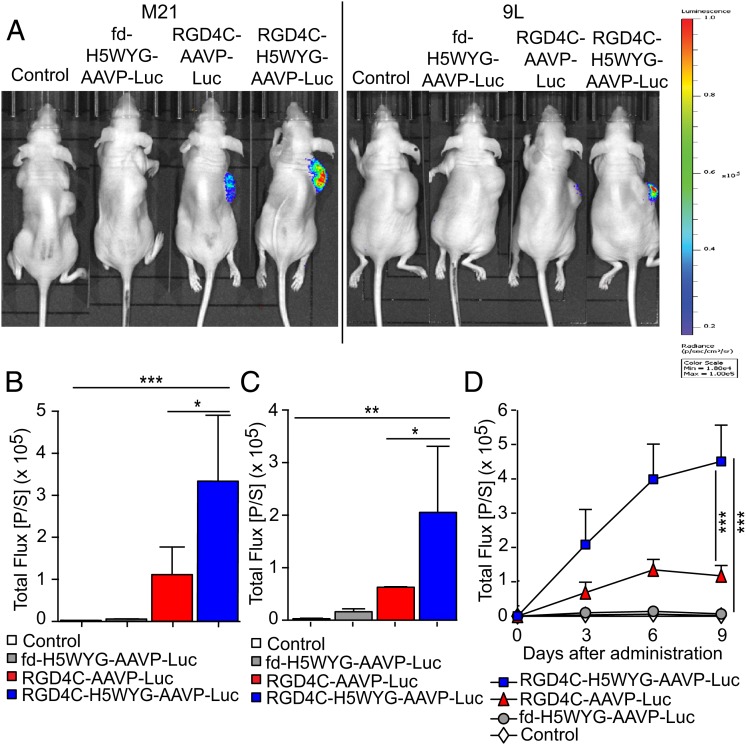
Systemic administration of the multifunctional RGD4C-H5WYG-AAVP-*Luc* mediates efficient gene delivery in tumors. (*A*) In vivo BLI in tumor-bearing mice. Nude mice with either M21 (*Left*) or 9L (*Right*) tumor xenografts received intravenous doses (5 × 10^10^ TU per mouse) of RGD4C-H5WYG-AAVP-*Luc*, RGD4C-AAVP-*Luc*, or nontargeted control fd-H5WYG-AAVP-*Luc*. Untreated mice were used as control. Representative mice are shown 6 d after vector administration. A standard calibration scale is provided. (*B* and *C*) Luciferase signals in M21 (*B*) and 9L (*C*) tumors quantified 6 d after vector delivery and expressed as total flux (p/s). (*D*) Serial real-time quantification of luciferase expression in M21 tumors. *P < 0.05; **P < 0.01; ***P < 0.001.

The original AAVP vectors were designed as targeted systemic gene therapy vectors for solid tumors in vivo (6–18). Many reports have demonstrated the ability of this vector to target and transduce tumors for therapeutic applications after intravenous administration without any gene expression detected in nontumor tissues (6, 8, 10–15). Because gene-therapy efficacy depends on therapeutic gene expression levels in vivo, we sought to investigate targeted gene delivery by RGD4C-H5WYG-AAVP to tumors in preclinical models. After confirming that the genomic alterations did not affect overall particle size (*SI Appendix*, Fig. S2), we used vectors carrying the *luciferase* reporter gene and assessed gene expression by bioluminescence imaging (BLI) (9, 10). We used immunodeficient nu/nu (nude) mice bearing subcutaneous tumors derived from M21 or 9L cells and administered RGD4C-H5WYG-AAVP-*Luc*, RGD4C-AAVP-*Luc*, or control nontargeted fd-H5WYG-AAVP-*Luc* vectors intravenously. Luciferase expression within M21 and 9L tumors was detectable 3 d after administration of either RGD4C-H5WYG-AAVP-*Luc* or RGD4C-AAVP-*Luc* particles. However, a notably higher level of luciferase was found in the tumors of the mice given RGD4C-H5WYG-AAVP-*Luc* (Fig. 6*A*). For example, at 6 d after AAVP administration, the boost in tumor luminescence from the multifunctional RGD4C-H5WYG-AAVP-*Luc* reached ∼3.2- and ∼3.5-fold higher than RGD4C-AAVP-*Luc* in mice bearing M21 (Fig. 6*B*) and 9L tumors (Fig. 6*C*), respectively. Moreover, BLI over time showed that gene expression by the original RGD4C-AAVP-*Luc* reached maximum gene expression and plateaued around 6 d after vector administration (Fig. 6*D*), consistent with previous reports (10). Alternatively, clearly higher tumor luciferase expression was achieved by RGD4C-H5WYG-AAVP-*Luc* at all time points, with a constant increase over time (Fig. 6*D*). No tumor-associated bioluminescent signals were observed with nontargeted control vectors, and no bioluminescence was observed in normal organs from all experimental groups, a result showing that systemic transgene delivery to tumors by the RGD4C-H5WYG-AAVP-*Luc* vector remained specific. Taken together, these data establish the superior gene delivery efficacy of this next-generation targeted AAVP vector in preclinical tumor models.

## Discussion

In this study, we designed, generated, and evaluated a next-generation of targeted AAVP constructs with enhanced abilities as gene delivery agents to cancer. To date, development of most multifunctional vectors still relies on synthetic conjugates introduced into genetic or molecular constructs (39). They typically are cumbersome and often system-specific, limiting broad applications. Many are unsuitable for clinical use due to poor biocompatibility and potential toxicity resulting from inorganic materials or surfactants (40). Several phage-based vectors are well suited for transgene delivery and can be conveniently engineered (2). Desired ligands (peptides, proteins or antibodies) can be displayed site-specific on the phage capsid simply by biosynthesis and self-assembly (41). Finally, phage can be produced under good manufacturing practice standards with simple, cost-effective, and scalable techniques. Since native phage particles cannot infect eukaryotic cells, and have a historic context as antibacterial agents, they are proven safe for human use (42), especially by the Food and Drug Administration as antibacterial food additives (43).

Here we explored how the capsid proteins of filamentous M13 phage, especially the multithousand copies of the major coat protein (pVIII) and the much smaller number of minor pIII protein copies on externally projecting filaments at one end of the phage particle, could be reengineered to display a wide range of functional peptide motifs with desirable attributes. Furthermore, incorporating ITR-flanked transgene expression cassettes from AAV vectors enables our multifunctional AAVP to transduce cancer cells in a targeted and specific manner (10). Thus, in this report, we have markedly improved the AAVP prototype to overcome inherent gene delivery challenges in mammals.

One substantially improved construct designed to escape a mammalian barrier is a multifunctional AAVP displaying the AKAS peptide on the phage surface, covered by thousands of pVIII protein moieties, to confer resistance to nonspecific plasma protein adsorption and antibody neutralization (29). We have shown that the introduction of the AKAS motif indeed neutralizes the negative charge of filamentous phage by generating a zwitterionic surface, thereby reducing fibrinogen adhesion and binding to positively charged molecules while avoiding antibody neutralization. This evasion of physiological barriers, although partial, should increase vector build-up within the tumor, subsequently promoting the existing in vivo efficacy of RGD4C-AAVP vector upon systemic administration. A key extracellular barrier to systemic phage-based gene delivery is fast removal of phage from the circulation after intravenous injection by reticulo-endothelial system (RES) macrophages (44). In general, nonspecific protein adsorption by circulating plasma proteins including fibrinogen, IgG, and complement factors (45, 46) is the first event that leads to an immune response. Since the lack of phage tropism to mammalian cells also means that the phage particles lack many of the pathogen-associated molecular patterns easily recognized by the mammalian immune system, the phage do not induce a robust immune response upon first contact (47); however, phage particles are not entirely ignored by the mammalian immune system, which will eventually sequester and clear them by the RES (48). We and other groups, reported that the immune response against phage does not hinder efficacy of repeated RGD4C-AAVP injections, but could instead enhance transduction of tumors (10, 12, 17). In human, a phage library based on the M13 phage (parent of the AAVP), was administered intravenously to cancer patients (49). No serious clinical side effects were observed with serial infusions, despite the presence of antiphage IgG, and phage were successfully recovered from tumors after repeated administrations. Recently, effective treatment of a 15-y-old cystic fibrosis patient with a disseminated *Mycobacterium abscessus* infection was reported by using a 3-phage mixture (50). Sera showed no evidence of phage neutralization.

Previously, we recognized that another major barrier to AAVP escape from endosomal vesicles was the acidic charge in lysosomes, and we had used a chemical disruptor, chloroquine *in trans*, to enable escape from endosomes (19). A better procedure is our design of multifunctional RGD4C-AAVP constructs that present endosomal escape peptides *in cis*, establishing that the vector displaying the histidine-rich H5WYG motif shows a marked increase in transgene delivery to cancer cells in vitro, as well as robust buffering capacity and improved systemic targeted gene delivery in solid tumors in vivo. Therefore, our next-generation multifunctional AAVP particles advance considerably the AAVP therapeutic efficacy against cancer. While this study yielded trifunctional AAVP vectors displaying H5WYG on the rpVIII or AKAS on the WT pVIII, a tetrafunctional AAVP could be designed for simultaneous display of both H5WYG and AKAS on the rpVIII and WT pVIII major coat proteins, respectively. Moreover, targeting ligands other than RGD4C can be used on the multifunctional AAVP to target further cancer cell types and other human diseases.

## Materials and Methods

Statistical analyses were done with Student’s *t* test or 1-way ANOVA with Tukey’s post hoc test. *P < 0.05, **P < 0.01, ***P < 0.001. All animal studies were first approved institutionally by the Animal Welfare and Ethical Review Body (AWERB), then by the Home Office.

Additional methods are detailed in the *SI Appendix*, *SI Materials and Methods*.

## Supplementary Material

Supplementary File
