# Supplementary material for: Next-generation of targeted AAVP vectors for systemic transgene delivery against cancer
Source: Proc Natl Acad Sci U S A. 2019 Aug 2;116(37):18571–7. doi: 10.1073/pnas.1906653116 (PMC6744886; doi:10.1073/pnas.1906653116)
Supplement: Supplementary File [file pnas.1906653116.sapp.pdf]

Supporting Information for:

Next-generation of targeted AAVP vectors for systemic transgene delivery against cancer

Keittisak Suwan, Teerapong Yata, Sajee Waramit, Justyna M. Przystal, Charlotte A. Stoneham, Kaoutar Bentayebi, Paladd Asavarut, Aitthiphon Chongchai, Peraphan Pothachareon, Koon-Yang Lee, Supachai Topanurak, Tracey L. Smith, Juri G. Gelovani, Richard L. Sidman, Renata Pasqualini, Wadih Arap, Amin Hajitou

Includes:

- Materials and Methods

- Fig. S1

- Fig. S1 Legend

- Fig. S2

- Fig. S2 Legend

Materials and Methods

Design and construction of the multifunctional bacteriophage

Multifunctional AAVP particles were made through a series of genetic modifications. The RGD4C.fUSE5 vector, in which the nucleotide sequence encoding the RGD4C peptide motif was fused in-frame with the pIII gene, was previously described in a detailed protocol (1). An f88-4 vector-derived DNA fragment containing a recombinant gene VIII (GenBank Accession Number: AF218363.1) was inserted in the RGD4C.fUSE5 backbone vector, and a mammalian transgene cassette was inserted in an intergenic region of the resultant vector. To display peptides

on the recombinant pVIII of RGD4C.fUSE5/f88-4, the corresponding nucleotide sequence and its complementary sequence were designed and 5′ phosphorylated. The phosphorylated oligos were mixed (20 pmol/primer), heated to 95˚C for 5 minutes and gradually cooled at room temperature to allow annealing. Annealing of sense and anti-sense oligonucleotides generates *Hind*III and *Pst*I sticky ends, ready to be cloned in the digested RGD4C.fUSE5/f88-4 phage plasmids. An aliquot of ligation reaction was transformed into DH5α competent cells and inoculated on LB agar plate containing 50 μg/ml tetracycline. Single colonies were picked and their plasmids were isolated with a QIAprep Spin Miniprep kit (QIAGEN). The positive clones were selected by sequencing analysis. To display peptides on the wild-type pVIII major coat protein, oligonucleotide sequences encoding a peptide of short length (up to 10 amino acid residues) were introduced into the wild-type VIII gene by using the Phusion Site-Directed Mutagenesis Kit. Briefly, the 50 μl polymerase chain reaction (PCR) reaction was performed with 10 pg template DNA, 0.5 μM 5′ phosphorylated primer pairs, 200 μM dNTP, and 0.02 U Phusion Hot Start II DNA polymerase. The reaction was initiated by pre-heating the reaction mixture to 98˚C for 30 seconds followed by 25 cycles of 98˚C for 15 seconds, 61˚C for 30 seconds, and 72˚C for 5 minutes. The PCR products were evaluated by agarose gel electrophoresis. The linear PCR product was circularized by ligation by using the T4 Quick ligase. Positive clones were selected with the same procedure as described above.

Production, purification, and titration

Targeted and control phage vectors were amplified and purified from the culture supernatant of host bacteria (*E. coli* K91Kan) as described in detail elsewhere (1). Because the recombinant gene pVIII in the chimera RGD4C-phage is transcribed from an IPTG-inducible *tac* promoter, we added 1 mM of IPTG (Isopropyl β-D-1-thiogalactopyranoside) during phage production to display the

peptide inserts. The titration of phage viral particles was carried out by infection of host *E.coli* K91Kan bacteria for colony counting and expressed as bacterial transducing units (TU). AAVP particles in suspension were sterile-filtered through 0.45-μm filters.

Immunofluorescence staining

Tumor cells were seeded on 18 mm^2 coverslips in 12-well plates and allowed to proliferate until 70-80% confluent. Cells were incubated with the AAVP particles for 4 hours, washed with PBS, and fixed in in PBS containing 4% PFA for 10 minutes at room temperature. Cells were treated with 50 mM ammonium chloride (NH_4Cl) to quench free aldehyde groups from fixation followed by permeabilization with 0.2% triton X-100. Cells were washed and blocked with PBS containing 2% BSA for 30 minutes. Cells were then incubated for 1 hour at room temperature with a rabbit anti-fd bacteriophage (diluted 1:1000). For secondary staining, cells were incubated with a goat anti-rabbit AlexaFluor-conjugated secondary antibody (diluted 1:750 in PBS containing 1% BSA) and with 4',6-diamidino-2-phenylindole (DAPI) (diluted 1:2,000 in PBS containing 1% BSA) for 1 hour at room temperature in the dark. Finally, cells were mounted in Mowiol mounting medium (Invitrogen) and images were obtained with a fluorescent microscope. Confocal images were acquired by using a Leica SP5 confocal microscope fitted with Argon, UV and HeNe lasers, with a 63x oil objective. Images were processed on ImageJ and Adobe Photoshop (Adobe Systems).

DEAE-dextran polymer assay

To coat the plate surface, 1 mL of DEAE-dextran 2mg/mL (Sigma) was incubated overnight at 37°C. The excess polymer was removed and washed once with 1ml PBS before addition of 5 μL of phage in 1 ml PBS and incubated overnight at 37°C. The phage suspension was recovered and

used to infect *E.coli* K91Kan for TU quantification.

Fibrinogen assay

Individual wells of a 48-well plate were coated with fibrinogen 2 mg/mL (Sigma) for 2 hours at 37°C. Excess fibrinogen was removed and 5 μL of phage in 110 μL serum-free media were added. After 1 hour incubation at 37°C, cell transduction was performed as described above in the cell transduction section. The levels of transduction were determined by luciferase assay.

Cell lines. The human M21 melanoma cells were from the American Type Culture Collection (ATCC), and the rat 9L glioblastoma cells were a gift from Dr. Hrvoje Miletic (University of Bergen, Norway). Both cell lines were maintained in a humidified incubator at 37°C in 5% CO_2 and cultured in Dulbecco's Modified Eagle's Medium (Sigma) supplemented with 10% fetal bovine serum (Sigma), penicillin (100 units/mL, Sigma), streptomycin (100 μg/mL, Sigma), and L-glutamine (2 mmol/L, Sigma).

Mammalian cell transduction by phage-based vectors and subsequent gene delivery

A subconfluent monolayer culture of cells was seeded in 48-well plates and incubated at 37°C for 24 hours, until 80% confluent. AAVP in serum-free medium was incubated with cells at 37°C with a ratio of 10^6 TU AAVP per cell, unless otherwise stated in the text. After 4 hours of incubation, complete medium was added to each well. The plate was incubated in CO_2 incubator at 37°C and the medium was renewed every day. Transgene expression was assessed at various time points depending on the experiment carried out. For luciferase assays, the Promega Steady-glo® luciferase assay kit was used to evaluate the gene expression in transduced cells. Luciferase

expression was quantified by using a Turner Biosystems microplate luminometer. Luciferase assays were performed in triplicate and normalized to 100 μg cell protein, as determined by the Bradford assay. Transduction of cells by using vector GC per cell was performed with a similar protocol. Calculation of vector titers as GC/cell was first done by using quantitative PCR of various vector dilutions.

Anti-phage antibody assay

Cells were treated as described above and transduced with a mixture of AAVP and an anti-phage polyclonal antibody (Sigma). The antibody was used at various dilutions and cell transduction was evaluated by the luciferase assay described above.

Endosome buffering capacity measurements

The acid-base titration method was used to determine the endosome buffering capacities of the multifunctional AAVP in sterile water. The pH was adjusted to 10 by adding NaOH, and subsequent additions of HCl were used to titrate the solution to pH 3, while changes in pH were recorded by using a pH meter. Titration of water was completed in parallel as a control. The natural endosome pH range of 7.0-4.0 was used to assess the endosome buffering capacity of the multifunctional AAVP.

Transmission electron microscopy of the AAVP particles

Carbon film-coated copper mesh grids were glow discharged to make the film surface hydrophilic, enabling better suspension of the AAVP particles on the grids. Particles from CsCl (Cesium Chloride)-purified samples were applied on the grids, left to incubate for 15 minutes, and removed

by blotting on absorbent paper. The grids were then washed with sterile-filtered deionized water, blotted on absorbent paper twice, and dried for 15 minutes. A solution of 1% uranyl acetate was applied to the grid for 30 seconds to negatively stain the particles. The grids were subsequently washed and blotted dry twice with sterile-filtered deionized water before being imaged using a scanning electron microscope (JEOL JEM-2010, UK) and analyzed by using ImageJ software.

Animal studies and *in vivo* bioluminescence imaging

To establish tumors in mice, 5×10^6 M21 or 2×10^6 9L cells were subcutaneously implanted into immunodeficient nude mice. Tumor-bearing mice received AAVP i.v. through the tail vein with vectors carrying the *luciferase* reporter gene at a dose of $5x10^{10}$ TU per mouse (1, 2). To monitor luciferase expression, mice were anesthetized and administered 100 mg/kg of d-luciferin (Gold Biotechnology), then imaged by using the *In Vivo* Imaging System (IVIS 100; Caliper Life Sciences). A region of interest was defined manually over the tumors to measure signal intensities recorded as total photon counts per second per cm^2 ($p/sec/cm^2/sr$) (1, 2). We used 5 mice per group and repeated the experiments twice. Because of the rapid growth of subcutaneous 9L tumors, which reach large size tumors quickly, we analyzed BLI over a shorter time course and in accordance to the "3Rs" (Reduce, Refine, and Replace) and Institutional and Home Office guidelines.

Fig. S1.

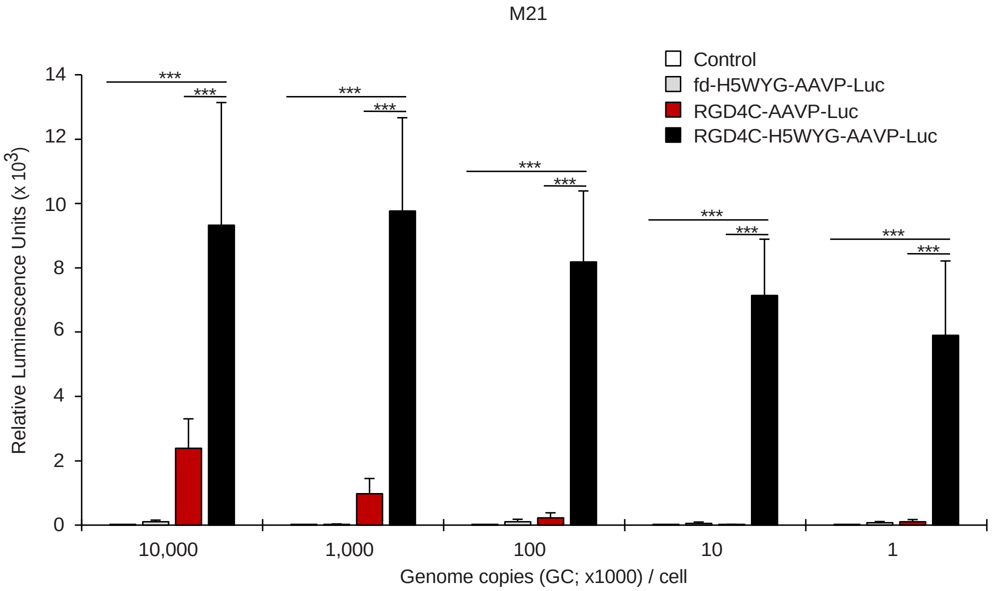

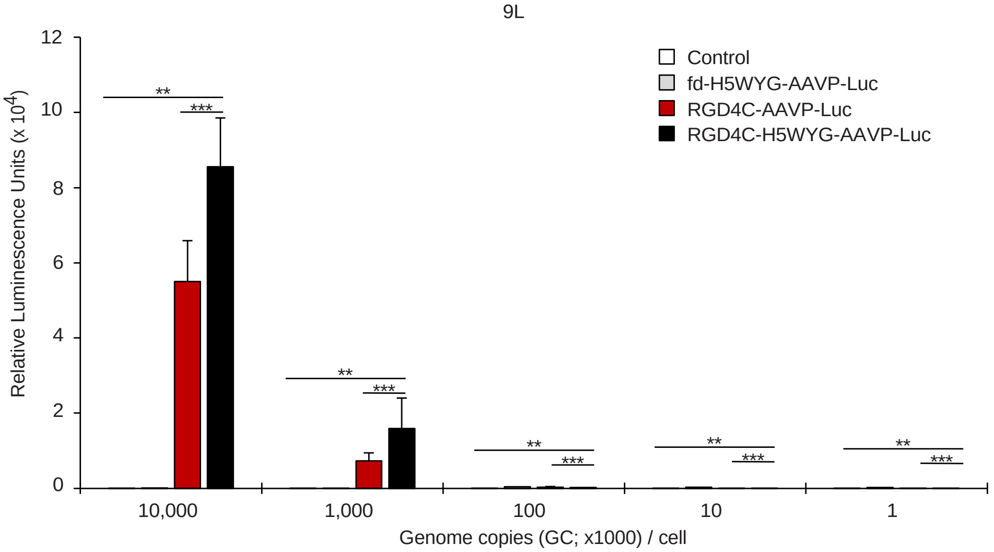

Fig. S1. Gene delivery by the multifunctional phage is dependent on vector genome copies. Quantification of luciferase expression at day 3 following transduction of M21 (top) and 9L (bottom) tumor cells with increasing genomes copies (GC)/cell of either RGD4C-H5WYG-AAVP-*Luc* or RGD4C-AAVP-*Luc***.** Non-targeted control fd-H5WYG-AAVP-*Luc* was also included. Untreated cells were used as control. **, p<0.01; ***, p<0.001

Fig. S2.

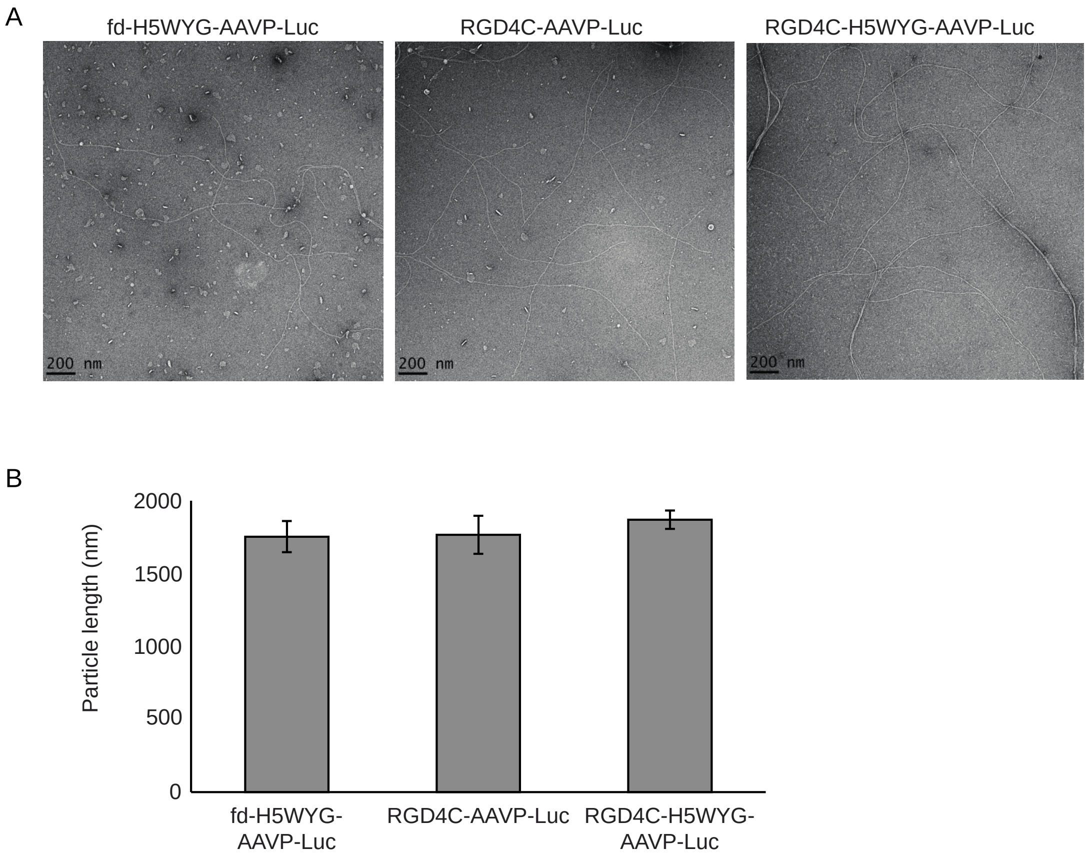

Fig. S2. (**A**) Transmission electronic microscopic (TEM) analysis of vectors**.** RGD4C-H5WYG-AAVP-*Luc*, RGD4C-AAVP-*Luc*, or non-targeted control (fd-H5WYG-AAVP-*Luc*) particles were characterized by negative staining and visualized by TEM. (**B**) Particle lengths are shown in nm. Scale bars, 200 nm.

References

1. A. Hajitou *et al.*, Design and construction of targeted AAVP vectors for mammalian cell transduction. *Nat. Protoc.* **2**, 523 (2007).

2. A. Hajitou *et al.*, A hybrid vector for ligand-directed tumor targeting and molecular imaging. *Cell* **125**, 385–398 (2006).
